# Supernatants from Water Extraction—Ethanol Precipitation of *Fagopyrum tararicum* Seeds Enhance T2DM Management in Mice by Regulating Intestinal Microbial Communities

**DOI:** 10.3390/foods15010143

**Published:** 2026-01-02

**Authors:** Xiaodong Ge, Xiaoxuan Du, Yaolin Wang, Yang Yang, Xiaoyu Gao, Yuchang Zhou, Yuting Jiang, Shiqi Xiao, Ligen Chen, Rong Shao, Wei Xu, Kyung-Min Kim, Na Wu

**Affiliations:** 1 College of Marine and Bioengineering, Yancheng Institute of Technology, Yancheng 224051, China; gexiaodongyg@163.com (X.G.); haobingshuaike@hotmail.com (X.D.); 19516690593@163.com (Y.W.); 18913064550@163.com (X.G.); 17372710809@163.com (Y.Z.); jyuting1120@163.com (Y.J.); 15896053836@163.com (S.X.); ycit549638894@outlook.com (L.C.); shaor1973@163.com (R.S.); xuweiyc@163.com (W.X.); 2 Jiangsu Key Laboratory for Exploration and Utilization of Marine Wetland Biological Resources, Yancheng Institute of Technology, Yancheng 224051, China; 3 Coastal Agriculture Research Institute, Kyungpook National University, Daegu 41566, Republic of Korea; 4 School of Chemistry & Chemical Engineering, Yancheng Institute of Technology, Yancheng 224051, China; 15189416922@163.com; 5 Department of Applied Biosciences, Kyungpook National University, Daegu 41566, Republic of Korea

**Keywords:** *Fagopyrum tararicum* seeds, type 2 diabetes *mellitus* mice, hyperglycemia, intestinal microbiota, short-chain fatty acids

## Abstract

Type 2 diabetes *mellitus* (T2DM) is an endocrine–metabolic disorder characterized by pancreatic islet dysfunction-induced hyperglycemia, which triggers hepatic injury, intestinal microbiota dysbiosis, and systemic complications. *Fagopyrum tararicum* seeds exhibit various biological activities, including antioxidant, hypolipidemic, and antihypertensive effects. However, there is limited research exploring how supernatants derived from the water extraction–ethanol precipitation of *Fagopyrum tararicum* seeds (SWEPFT) modulate the intestinal microbiota and their potential link to T2DM. This study evaluates SWEPFT’s effects on hyperglycemia and intestinal microbiota in T2DM mice. After a 4-week therapeutic period, SWEPFT markedly ameliorated hyperglycemia, as evidenced by reduced body weight (BW), fasting blood glucose (FBG), and glycated serum protein (GSP) and improved insulin sensitivity/resistance indicators (HOMA-IS/IR) and β-cell function (HOMA-β). Furthermore, the levels of both *Akt1* and *Slc2a2* transcription displayed notable enhancement. SWEPFT-H (high-dose SWEPFT) exhibited superior effects to SWEPFT-L (low-dose SWEPFT) in improving BW, FBG, and HOMA-IS. Moreover, SWEPFT modulated the intestinal microbiota by decreasing the Firmicutes/Bacteroidetes ratio, augmenting the proportion of *Intestinimonas* and *Ruminiclostridium*, and increasing the short-chain fatty acid content. A correlation analysis identified *Candidatus_Arthromitus*, *Anaeroplasma*, *Candidatus_Stoquefichus*, and *Harryflintia* as potential T2DM biomarkers linked to glycemic regulation. These findings elucidate SWEPFT’s critical role in microbiota modulation and hyperglycemia alleviation, providing a novel perspective for T2DM pathogenesis research and therapeutic development.

## 1. Introduction

Diabetes *mellitus* is a metabolic condition characterized by elevated blood glucose levels stemming from impaired insulin production or functionality [1]. Based on data from the International Diabetes Federation (IDF), about 537 million adults globally experience diabetes *mellitus*, with China recording 140.9 million instances, ranking highest internationally [2]. The IDF anticipates that the number of diabetes *mellitus* cases will rise to 783 million by 2045. The disease manifests mainly as type 1 diabetes *mellitus* (T1DM), a condition requiring insulin due to inadequate hormone production, and type 2 diabetes *mellitus* (T2DM), which emerges predominantly from reduced insulin effectiveness in peripheral body tissues [3]. T2DM constitutes over 90% of documented diabetes *mellitus* cases. Chronic glucose metabolism dysregulation in T2DM leads to abnormal oxidative stress, resulting in severe damage to the tissue microenvironment, particularly in the liver, pancreas, and intestines. This, in turn, can cause irreversible complications, including diabetic nephropathy, uremia, and diabetic cardiomyopathy, imposing significant physical, emotional, and economic burdens on patients [4]. Therefore, addressing hyperglycemia in T2DM is crucial for improving quality of life and safeguarding public health in China.

The etiology and pathogenesis of T2DM are multifactorial and complex [5]. Current research demonstrates a robust link between intestinal microbiota and the onset and progression of T2DM [6]. At the phylum level, Bacteroidetes and Firmicutes dominate the gut microbiota, accounting for over 95% of the microbial population. Imbalances in this ratio, particularly an elevation in the ratio of Firmicutes to Bacteroidetes (Firmicutes/Bacteroidetes), have been linked to body weight (BW) changes, including obesity [7]. Consequently, effective BW management is closely linked to the gut microbiota composition [8]. *Prevotella*, a prevalent symbiotic bacterium, produces anti-inflammatory factors that boost tight junction protein levels, subsequently strengthening intestinal barrier function in individuals with T2DM [9]. *Akkermansia muciniphila*, a mucin-degrading bacterium found in the intestinal mucus layer, modulates intestinal inflammation in T2DM by regulating microbiota translocation. In fact, the abundance of *Akkermansia muciniphila* has been found to correlate with improved glucose homeostasis [10]. Importantly, the gut microbiota influence glucose control and insulin sensitivity through the fermentation of fiber-rich nutrients to generate short-chain fatty acids (SCFAs). Current hypoglycemic medications, such as acarbose, metformin, sulfonylureas, thiazolidinediones, and DPP-4 inhibitors [11], improve blood glucose control by inhibiting glucosidase activity, activating AMPK or PPARγ, or inhibiting DPP-4. However, these drugs are not designed to regulate the intestinal microbiota, and some of them (such as acarbose) often cause adverse gastrointestinal reactions such as abdominal distension and diarrhea [12]. It is worth noting that intestinal microbiota imbalance itself is one of the core pathological features of T2DM. Its characteristics include a decrease in the abundance of SCFA-producing bacteria, a decline in SCFA levels, and impaired intestinal barrier function. Existing therapies have a limited ability to reverse this pathological state. Therefore, the discovery of active compounds in natural plants that can effectively decrease the level of blood glucose, regulate the intestinal microbiota, increase SCFA levels, and reduce adverse reactions has become a highly promising research direction.

*Fagopyrum tataricum* (*F. tataricum*), commonly known as *Tartary buckwheat*, is an annual dicotyledonous herb in the *Fagopyrum* genus of the Polygonaceae family. Studies have demonstrated that *F. tataricum* exhibits a range of biological activities, including antidiabetic, antihypertensive, and hypolipidemic effects, and it holds potential for preventing and improving chronic diseases [13]. Studies examining *F. tataricum* have primarily concentrated on polysaccharide isolation methods and their biological impacts. For instance, Ren et al. isolated a specific polysaccharide component from *Tartary buckwheat* root (TBRP-1) and investigated its hypoglycemic properties [14]. Their findings demonstrated that TBRP-1 markedly decreased blood glucose concentrations, glycated hemoglobin levels, and hepatic lipid content in T2DM mice, while enhancing insulin receptor substrate-1 and glucose transporter type 4 protein expression, consequently improving insulin sensitivity. Notably, in the water extraction–ethanol precipitation method for preparing *F. tataricum* polysaccharide, the polysaccharide is collected after precipitation and freeze-drying, while the supernatant is discarded [15]. Geng et al. reported that *F. tararicum* seeds, used as an edible grain crop in China, are rich in nutrients and functional components, and are recommended as a healthy cereal food [16]. In addition, compared with the whole seed powder, active components such as flavonoids and isoflavonoids were significantly enriched in the supernatant obtained from water extraction–ethanol precipitation of *F. tataricum* seeds (SWEPFT). These high-concentration active ingredients may be more easily recognized and utilized by the intestinal microbiota, thereby demonstrating stronger effects in regulating the microbial community and improving insulin resistance and liver lipid metabolism than a daily dietary dose of whole seed powder [17]. Moreover, the extraction process releases the active ingredients from the complex plant cell walls and dietary fiber matrix, thereby significantly enhancing the efficiency of their interaction with the intestinal microbiota. Nevertheless, limited research has investigated how SWEPFT influences gut microbiota in T2DM mice and its possible link to T2DM. This investigation seeks to develop a T2DM mouse model for evaluating both the hypoglycemic capabilities and the modulatory impact of SWEPFT on intestinal microbial populations in T2DM mice. This investigation establishes groundwork for examining the hypoglycemic properties of SWEPFT and presents a theoretical framework for maximizing the value of *F. tataricum*.

## 2. Materials and Methods

### 2.1. SWEPFT Preparation

Pulverized *F. tataricum* seeds (Xichang Three: Tartary Buckwheat Development Co., Ltd., Xichang, China) were passed through a 40-mesh screen after grinding. The sieved substance underwent extraction by soaking in deionized water (50:1, *v*/*w*) with reflux processing at 70 °C for 5 h. After extraction, the liquid portion underwent filtration, vacuum-based concentration, and lyophilization to generate SWEPFT for animal testing purposes. Samples were analyzed using UPLC-QTOF-MS/MS (Thermo Fisher Scientific, Waltham, MA, USA) with a Waters ACQUITY UPLC BEH Amide column (1.7 μm, 2.1 mm × 100 mm). The column temperature was set to 25 °C, flow rate at 0.5 mL/min, and injection volume at 2 μL. The mobile phases consisted of A (water with 25 mM ammonium acetate and 25 mM ammonia) and B (acetonitrile). The gradient elution program was as follows: 0–0.5 min, 95% B; 0.5–7 min, linear decrease from 95% to 65% B; 7–8 min, linear decrease from 65% to 40% B; 8–9 min, held at 40% B; 9–9.1 min, linear increase from 40% to 95% B; 9.1–12 min, held at 95% B.

### 2.2. Animal Experiment

Fifty healthy male ICR mice (SPF, 4 weeks old, weighing 20 ± 3 g) were procured from the Comparative Medical Center of Yangzhou University. The mice were maintained in an environment-controlled barrier facility (24 ± 2 °C) under regulated temperature and humidity conditions with alternating 12 h light/dark periods. The mice had unlimited access to food and water to sustain normal physiological functions. Following a week-long adaptation period, 10 specimens were arbitrarily split into the Normal cohort and provided conventional laboratory chow, whereas the remaining animals consumed a high-sugar and high-fat diet (HSHF; comprising 58.8% basic feed, 15% sucrose, 15% lard, 1% cholesterol, 0.2% cholate, and 10% egg yolk) to induce insulin resistance. After 4 weeks, the 40 animals receiving the HSHF diet underwent intraperitoneal administration of streptozotocin (STZ) to partially destroy the function of pancreatic β-cells. STZ dissolved in 0.1 M citrate buffer (pH 4.5) at 35 mg/kg was administered thrice weekly. The Normal cohort received equivalent citrate buffer injections. A period of 48 h after the third STZ injection, all mice underwent 12 h of fasting, and blood specimens were obtained via tail vein puncture for fasting blood glucose (FBG) determination. Mice exhibiting FBG levels ≥ 11.1 mM were classified as having established T2DM. These T2DM mice were subsequently arbitrarily split into four cohorts: the Model cohort (*n* = 10), low-dose SWEPFT cohort (SWEPFT-L, 100 mg/kg/d, *n* = 10), high-dose SWEPFT cohort (SWEPFT-H, 300 mg/kg/d, *n* = 10), and metformin hydrochloride cohort (MET, 100 mg/kg/d, *n* = 10). Treatment cohorts underwent daily oral gavage for 4 weeks per their assigned dosage. The Normal and Model cohorts were administered deionized water at 100 mg/kg/d. During the treatment phase, all mice retained unrestricted access to food and water. Mouse health conditions were observed daily, with BW and FBG measurements recorded weekly. After 4 weeks of intervention, an oral glucose tolerance test (OGTT) was executed. FBG was measured initially (G_0h_), and blood glucose concentrations were assessed at 0.5 h, 1 h, and 2 h after oral delivery of a 2 g/kg BW glucose solution (G_0.5h_, G_1h_, and G_2h_). The area under the curve (AUC) for OGTT was computed, utilizing AUC = 0.25 × (G_0h_ + G_0.5h_) + 0.25 × (G_0.5h_ + G_1h_) + 0.5 × (G_1h_ + G_2h_). On the subsequent day, the mice were anesthetized utilizing a combination of Sutazine (55 mg/kg BW) and Cyrazine hydrochloride (5 mg/kg BW). Blood specimens were collected via orbital sampling, after which mice were humanely euthanized through cervical dislocation. The liver sections and cecal material were extracted. Selected liver fragments were preserved in 4% paraformaldehyde solution for histological examination, whereas additional samples underwent rapid freezing utilizing liquid nitrogen and were maintained under ultra-low-temperature storage for future analytical procedures. The animal experimental protocols adhered to NIH guidelines concerning laboratory animal care and usage (NIH Publication No. 85-23 Rev. 1985), with procedural approval granted by the Lab Animal Ethical Committee of Jiangsu Vocational College of Medicine (approval number: SYLL-2023-704).

### 2.3. Homeostatic Model Assessment (HOMA) Insulin Correlation Indexes

The blood samples were kept at room temperature for 2 h, subsequently undergoing centrifugation at 447× *g* for 15 min; thereafter, the supernatant (serum) was procured. Fasting insulin (FINs) serum concentration was assessed utilizing an ELISA kit (Wuhan Chundu Biotechnology Co., Ltd., Wuhan, China) per the supplier’s protocol. Pancreatic islet function was assessed using HOMA indices, including the HOMA-β (β-cell function index: 20 × FINs/(FBG − 3.5)), HOMA-IR (insulin resistance index: FINs × FBG/22.5), and HOMA-IS (insulin sensitivity index: 1/(FINs × FBG)) [18].

### 2.4. Examination of Serum and Liver Biochemical Indexes

Blood serum specimens were processed according to Section 2.3. The liver specimens underwent mechanical disruption in physiological saline solution, followed by centrifugation at 1789× *g* for 15 min. After processing, the upper liquid phase was extracted to examine biochemical indicators, encompassing TC, TG, LDL-c, and HDL-c [19]. These parameters were determined per the protocols provided with biochemical kits procured from Nanjing Jiancheng Bioengineering Institute (Nanjing, China).

### 2.5. qPCR

Liver tissue specimens were removed from an ultra-low-temperature freezer, and a 0.1 g sample was taken and pulverized into powder before being placed into a 1.5 mL centrifuge tube. RNA was isolated employing TRIzol reagent (Invitrogen, Carlsbad, CA, USA) and the RevertAid First Strand cDNA Synthesis Kit (Takara, Kusatsu, Japan) was employed for cDNA preparation. Real-time Quantitative PCR (qPCR) analysis was executed with SYBR^®^ Premix Ex Taq™ II (Takara, Kusatsu, Japan) on an ABI 7500 real-time PCR instrument (Applied Biosystems, Foster City, CA, USA). The analysis utilized *Actb* as the housekeeping gene, while specific primers were constructed for serine/threonine-protein kinase 1 (*Akt1*) and solute carrier family 2 member 2 (*Slc2a2*) (Table 1). Thermal cycling parameters included initial denaturation (95 °C, 5 min), succeeded by 40 cycles alternating between 95 °C (15 S) and 65 °C (60 S), concluding with a final extension phase (65 °C, 5 min). Relative mRNA expression was quantified employing the 2^−ΔΔCt^ methodology.

### 2.6. Examination of Cecal Microbiota

Cecal contents (0.1 g) were obtained, and metagenomic DNA extraction was carried out per the protocol established by Ge et al. [20], utilizing universal primers 341F (“5′-CCTAYGGGRBGCASCAG-3′”) and 806R (“5′-GGACTACNNGGGTATCTAAT-3′”) for amplification. The sequencing procedure was executed utilizing the Illumina NovaSeq PE250 platform (Novogene Co., Ltd., Beijing, China). Raw sequencing data underwent merging and quality filtering steps to yield clean datasets; subsequently, OTU (Operational Taxonomic Unit) clustering and taxonomic classification analysis were implemented. The sequence analysis followed protocols established by Huang et al. [21].

### 2.7. Assessment of SCFAs in Cecum

Individual standard solutions of acetic, propionic, isobutyric, butyric, isovaleric, and valeric acid were formulated by diluting 100 μL of each acid in a 10 mL volumetric flask, and subsequently adding anhydrous ether until the designated volume was reached, establishing a composite standard stock solution. This solution was further diluted to six standard concentrations using anhydrous ether in a double gradient for gas chromatographic analysis. The cecal contents were homogenized with deionized water (1:10, *w*/*v*), followed by the addition of phosphoric acid and vortexing. SCFAs were extracted from the cecum samples using anhydrous ether and quantified by gas chromatography (GC-2010Plus, Shimadzu, Kyoto, Japan), utilizing a 0.22 μm nylon 6 filter. Chromatographic parameters were as follows: injector temperature 260 °C, sample volume 1 μL, split ratio 10:1, analytical column: HP-INNOWAX capillary column (30 m × 0.25 mm × 0.25 μm), flow rate 1 mL/min. High-purity nitrogen was utilized as the carrier gas. The thermal program was initiated at 100 °C (1 min hold) and ramped up to 200 °C at a rate of 5 °C/min with a final hold of 2 min.

### 2.8. Statistical Analysis

All experimental data are depicted as the mean ± standard deviation (SD). For statistical analysis, the Kruskal–Wallis test coupled with Dunn’s post hoc test incorporating Bonferroni’s correction (SPSS 16.0) were employed. Statistical significance was developed at *p* < 0.05. All statistical analyses were performed using the SPSS Statistics (version 26.0) software (IBM, Armonk, NY, USA) and GraphpadPrism (version 7.0, GraphpadSoftware, Inc., San Diego, CA, USA). Microbial abundance at phylum and genus levels among all groups was compared based on the Kruskal–Wallis test with false discover rate (FDR). Spearman’s rank or Pearson’s correlation was used to analyze the correlation between the relative abundance of gut microbiota and hypoglycemic parameters.

## 3. Results

### 3.1. Composition of SWEPFT

In this investigation, 4.72 g of SWEPFT was procured from 100 g of dried *F. tataricum* seeds. Through the UPLC-QTOF-MS/MS analysis, several essential bioactive constituents were detected, encompassing flavonoids (25), such as nobiletin, 5-hydroxyflavone, glabrol, and poncirin; isoflavonoids (14), including mefenamic acid, formononetin, glabridin, and daidzein; phenols (53), such as sinapyl alcohol, 3-hydroxyphenylacetic acid, homogentisic acid, and dobutamine; and indoles and derivatives (55), including indole-3-carboxaldehyde, indolelactic acid, and bufotenine (Table S1).

### 3.2. Effects of SWEPFT on BW, FBG, and AUC of OGTT and Serum GSP Levels in Mice

**Figure 1:**
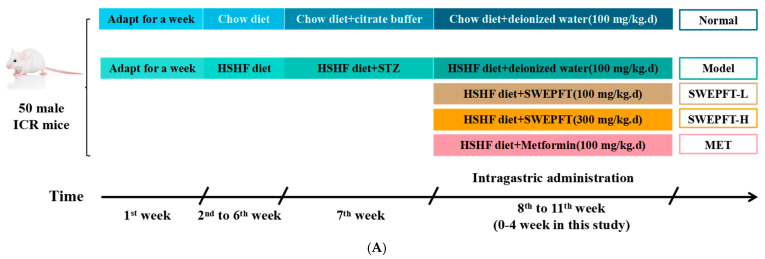

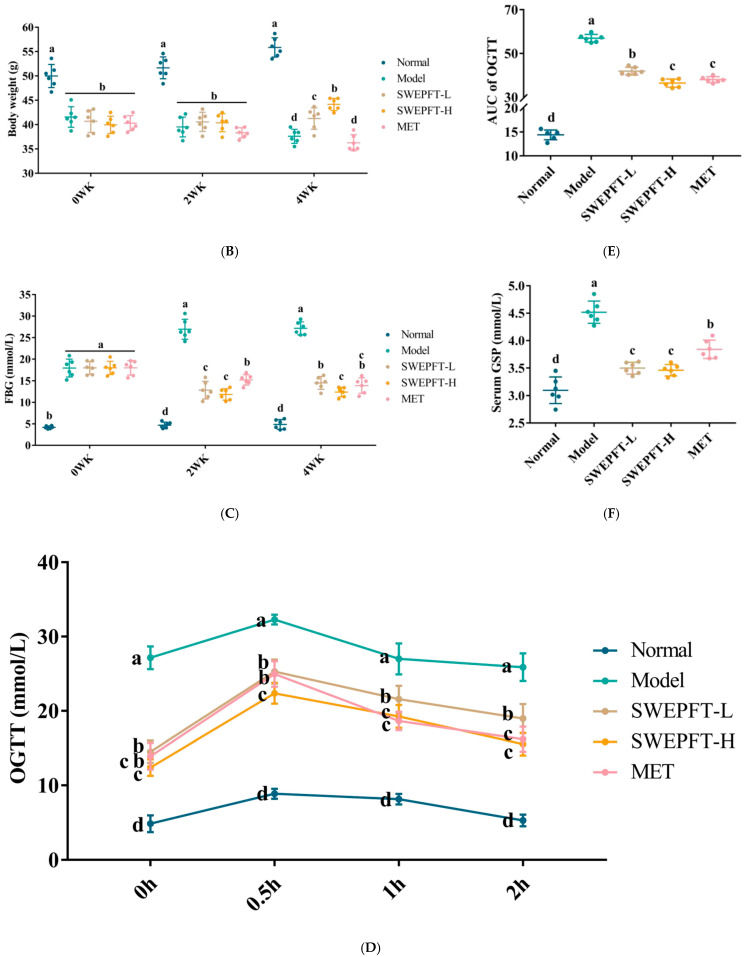
(**A**) The timeline of animal experiment; Effects of SWEPFT treatment during the experimental period: (**B**) Body weight, (**C**) FBG, (**D**) OGTT, (**E**) AUC of OGTT, and (**F**) serum GSP. SWEPFT refers to supernatants from the water extraction-ethanol precipitation of *Fagopyrum tararicum*, and T2DM indicates type 2 diabetes mellitus. WK stands for week. Different letters represent statistically significant differences between groups (*p* < 0.05).

Figure 1A displays the timeline of the animal experiments. As illustrated in Figure 1B, at baseline (week 0), excluding the Normal cohort, the BW in all cohorts was markedly diminished (*p* < 0.05). After a 2-week SWEPFT intervention, no marked alterations in BW were observed across the cohorts (*p* > 0.05). Nevertheless, after 4 weeks, BW in all treatment cohorts remained markedly diminished compared to that in the Normal cohort (*p* < 0.05). Notably, the BW in the SWEPFT-L and SWEPFT-H cohorts was markedly elevated compared to that in the Model and MET cohorts (*p* < 0.05). Figure 1C illustrates the trends in FBG levels across the cohorts, showing that, at the initial phase (week 0), FBG in the experimental cohorts exhibited markedly elevated values compared to those in the Normal cohort (*p* < 0.05). After two weeks of SWEPFT administration, FBG values across all treatment cohorts demonstrated a substantial reduction relative to the Model cohort (*p* < 0.05), wherein the SWEPFT-L and SWEPFT-H cohorts displayed notably decreased FBG measurements versus the MET cohort (*p* < 0.05). Upon completion of four weeks of treatment, the FBG readings in the SWEPFT-L and SWEPFT-H cohorts remained distinctly elevated compared to those in the Normal cohort (*p* < 0.05) yet were considerably reduced compared to the Model cohort (*p* < 0.05). Notably, the FBG measurements in the SWEPFT-H cohort were lower than those observed in the SWEPFT-L cohort (*p* < 0.05).

Figure 1D illustrates the glucose tolerance capacity exhibited by the T2DM mice. Following gastric delivery of glucose solution (2 g/kg.BW), the glucose concentrations initially spiked before showing a progressive decline in all cohorts. Before glucose administration (0 h), the SWEPFT-L, SWEPFT-H, and MET cohorts displayed glucose levels markedly elevated compared to the Normal cohort (*p* < 0.05), yet substantially below the Model cohort (*p* < 0.05). Glucose levels peaked at 0.5 h across the cohorts, with the SWEPFT-L, SWEPFT-H, and MET cohorts demonstrating markedly reduced levels compared to the Model cohort (*p* < 0.05). The SWEPFT-H cohort exhibited particularly notable reductions compared to both the SWEPFT-L and MET cohorts (*p* < 0.05). During the 1 h and 2 h intervals, glucose concentrations demonstrated a steady reduction, with the SWEPFT-H and MET cohorts showing marked decreases compared to the SWEPFT-L cohort (*p* < 0.05). The OGTT AUC, depicted in Figure 1E, revealed significant reductions across the treatment cohorts compared to the Model cohort (*p* < 0.05), with the SWEPFT-H and MET cohorts demonstrating substantially lower values than those of the SWEPFT-L cohort (*p* < 0.05). As depicted in Figure 1F, the GSP measurements indicated significant reductions in the Normal, SWEPFT-L, SWEPFT-H, and MET cohorts compared to the Model cohort (*p* < 0.05). Additionally, the SWEPFT-L and SWEPFT-H cohorts demonstrated markedly reduced GSP levels versus the MET cohort (*p* < 0.05).

### 3.3. Effects of SWEPFT on Insulin Correlation Indexes and Serum Biochemical Indicators

**Figure 2:**
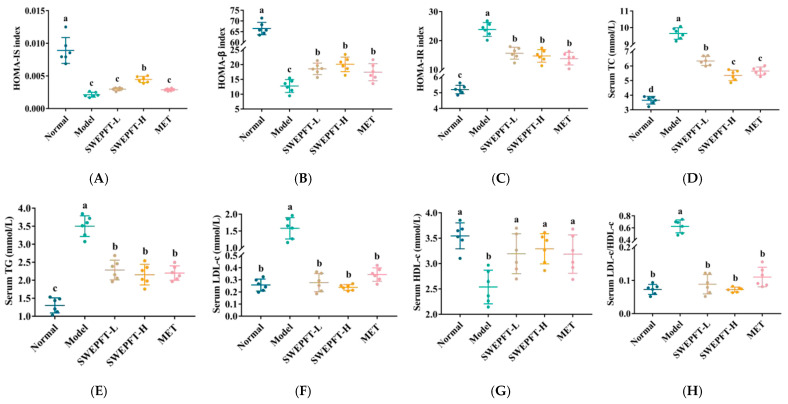
Pancreatic islet function correlation indices and serum biochemical indicators: (**A**) HOMA-IS, (**B**) HOMA-β, (**C**) HOMA-IR, (**D**) serum TC, (**E**) serum TG, (**F**) serum LDL-c, (**G**) serum HDL-c, and (**H**) serum LDL-c/HDL-c. Different letters represent statistically significant differences between groups (*p* < 0.05).

As depicted in Figure 2A, the HOMA-IS levels were markedly diminished in all cohorts compared to those in the Normal cohort (*p* < 0.05). Notably, HOMA-IS in the SWEPFT-H cohort was markedly elevated compared to those in the Model, SWEPFT-L, and MET cohorts (*p* < 0.05). Figure 2B demonstrates that there was a considerable reduction in the HOMA-β in the SWEPFT-L, SWEPFT-H, and MET cohorts versus that in the Normal cohort (*p* < 0.05), which maintained elevated levels compared to the Model cohort (*p* < 0.05). The HOMA-IR measurements in the SWEPFT-L, SWEPFT-H, and MET cohorts revealed substantial decreases compared to the Model cohort (*p* < 0.05), while remaining elevated versus the Normal cohort (*p* < 0.05) (Figure 2C). T2DM affects not only blood glucose but also various serum biochemical parameters. Figure 2D indicates that all cohorts exhibited substantial decreases in serum TC compared to that in the Model cohort (*p* < 0.05), with the SWEPFT-H and MET cohorts depicting marked reductions versus the SWEPFT-L cohort (*p* < 0.05), but these were elevated compared to the Normal cohort (*p* < 0.05). According to Figure 2E, the SWEPFT-L, SWEPFT-H, and MET cohorts displayed significant decreases in serum TG measurements compared to those in the Model cohort (*p* < 0.05), while these were elevated compared to those in the Normal cohort (*p* < 0.05). Figure 2F demonstrates that the serum LDL-c exhibited marked decreases across all cohorts compared to the Model cohort (*p* < 0.05), with comparable levels among the SWEPFT-L, SWEPFT-H, MET, and Normal cohorts (*p* > 0.05). The serum HDL-c measurements showed substantial increases across all cohorts compared to the Model cohort (*p* < 0.05), maintaining similar levels among the SWEPFT-L, SWEPFT-H, MET, and Normal cohorts (*p* > 0.05) (Figure 2G). Regarding the LDL-c/HDL-c ratio, an essential indicator of metabolic disorder risk, Figure 2H indicates substantial decreases across all cohorts compared to that in the Model cohort (*p* < 0.05).

### 3.4. Effects of SWEPFT on Biochemical Indicators and Key Genes in the Liver

**Figure 3:**
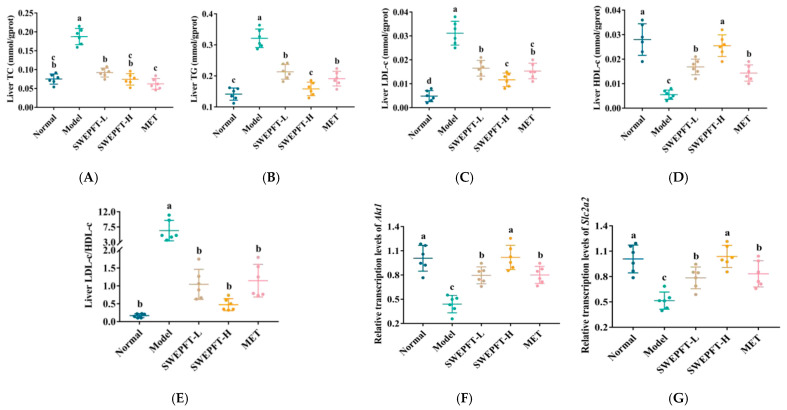
Biochemical indicators and histopathological analysis of liver tissue: (**A**) Liver TC, (**B**) liver TG, (**C**) liver LDL-c, (**D**) liver HDL-c, (**E**) liver LDL-c/HDL-c, and the effects of SWEPFT on mRNA transcription levels: (**F**) *Akt1* and (**G**) *Slc2a2*. Different letters represent statistically significant differences between groups (*p* < 0.05).

Lipid metabolism disorder is a multifaceted pathological process that involves lipid synthesis, breakdown, and absorption within the body. When these processes are disrupted, particularly due to enhanced lipid synthesis in the liver, lipid accumulation occurs, leading to inflammatory responses, hepatocellular injury, and, ultimately, threats to overall health. As depicted in Figure 3A–C, the liver TC, TG, and LDL-c levels were markedly diminished in all of the treatment cohorts compared to those in the Model cohort (*p* < 0.05). Specifically, the liver TG level in the SWEPFT-H cohort was markedly diminished compared to that in the SWEPFT-L and MET cohorts (*p* < 0.05) (Figure 3B), and the liver LDL-c level in the SWEPFT-H cohort was markedly diminished compared to that in the SWEPFT-L cohort (*p* < 0.05) (Figure 3C). In contrast, the liver HDL-c levels in the Normal, SWEPFT-L, SWEPFT-H, and MET cohorts were markedly elevated compared to those in the Model cohort (*p* < 0.05) (Figure 3D), with the SWEPFT-H cohort displaying markedly elevated levels compared to the SWEPFT-L and MET cohorts (*p* < 0.05). Based on Figure 3E, the liver LDL-c/HDL-c proportions diminished substantially across all of the treated cohorts compared to those in the Model cohort (*p* < 0.05). The data presented in Figure 3F,G indicate that the *Akt1* and *Slc2a2* transcription levels were notably elevated in the Normal, SWEPFT-L, SWEPFT-H, and MET cohorts compared to those in the Model cohort (*p* < 0.05). Moreover, the analysis revealed that the SWEPFT-H treatment resulted in markedly enhanced *Akt1* and *Slc2a2* transcription when evaluated against both the SWEPFT-L and MET treatments (*p* < 0.05).

### 3.5. Effects of SWEPFT on Intestinal Microbial Communities and Short-Chain Fatty Acids in Cecum Contents

**Figure 4:**
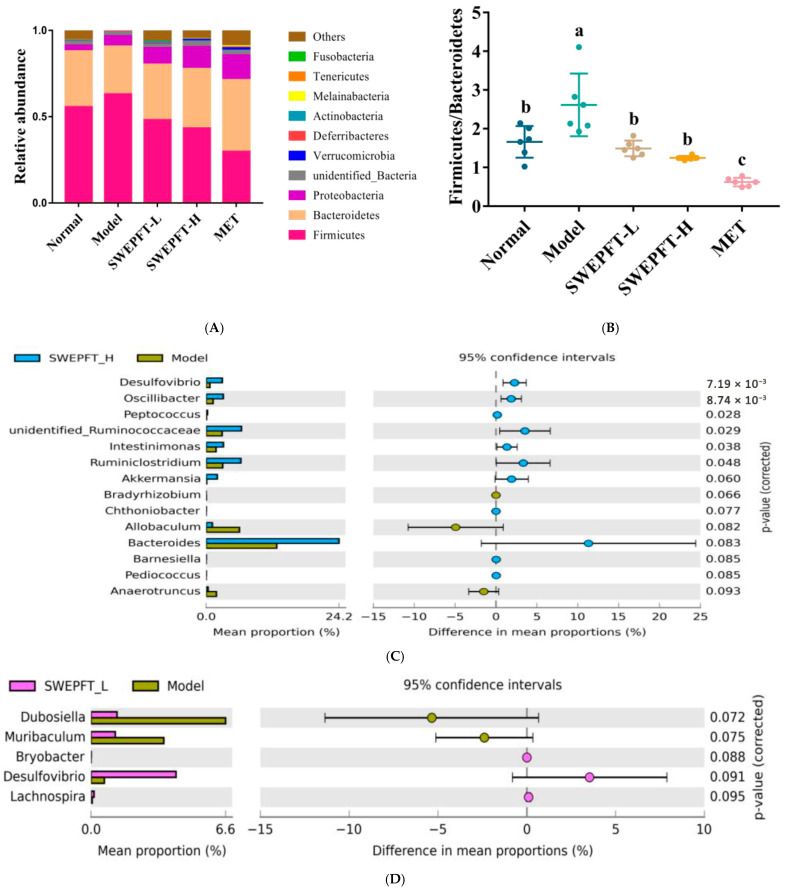
Effects of SWEPFT treatment on intestinal microbial communities in the cecum: (**A**) diversity of gut microbiota at the phylum level, (**B**) Firmicutes/Bacteroidetes ratio. Extended error bar plot showing significant differences in intestinal microbiota at the genus level: (**C**) SWEPFT-H vs. Model; (**D**) SWEPFT-L vs. Model. Different letters represent statistically significant differences between groups (*p* < 0.05).

**Figure 5:**
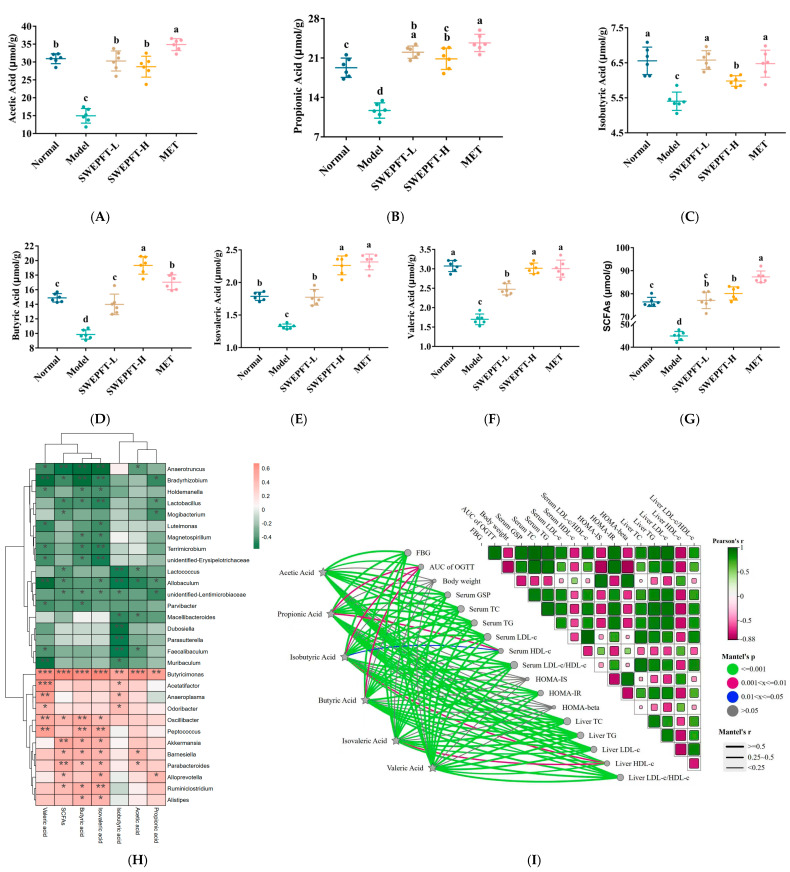
Effects of SWEPFT treatment on SCFAs content in the cecum: (**A**) Acetic acid, (**B**) Propionic acid, (**C**) Isobutyric acid, (**D**) Butyric acid, (**E**) Isovaleric acid, (**F**) Valeric acid, (**G**) Total SCFAs. (**H**) Spearman correlation heat map between bacterial genera and SCFAs. (**I**) Mantel test analysis showing the correlation between hypoglycemic parameters and SCFAs. Different letters represent statistically significant differences between groups (*p* < 0.05). * *p* < 0.05, ** *p* < 0.01, and *** *p* < 0.001 indicate the significant relationship between bacterial genera and SCFAs.

Within gut microbiota, Firmicutes and Bacteroidetes represent the predominant phyla, with their ratio demonstrating a strong correlation to hyperglycemia in T2DM mice. Figure 4A indicates that the Normal, SWEPFT-L, SWEPFT-H, and MET cohorts exhibited a diminished Firmicutes abundance alongside elevated Bacteroidetes levels when compared to the Model cohort. Figure 4B demonstrates that these cohorts displayed markedly reduced Firmicutes/Bacteroidetes ratios compared to those in the Model cohort (*p* < 0.05). A genus-level examination revealed distinct bacterial community variations between the SWEPFT-H, SWEPFT-L, and Model cohorts. Figure 4C illustrates bacterial genus modifications in the SWEPFT-H cohort compared to those in the Model cohort. Specifically, *Desulfovibrio*, *Oscillibacter*, *Peptococcus*, *Unidentified-Ruminococcaceae*, *Intestinimonas*, *Ruminiclostridium*, *Akkermansia*, *Chthoniobacter*, *Bacteroides*, *Barnesiella*, and *Pediococcus* were more abundant, while *Bradyrhizobium*, *Allobaculum*, and *Anaerotruncus* showed decreased relative abundances. As illustrated in Figure 4D, *Bryobacter*, *Desulfovibrio*, and *Lachnospira* were more abundant in the SWEPFT-L cohort, whereas *Dubosiella* and *Muribaculum* were reduced compared to the Model cohort. Interestingly, the *Desulfovibrio* abundance increased in both the SWEPFT-H and SWEPFT-L cohorts compared to that in the Model cohort. SCFAs, encompassing acetic acid, propionic acid, isobutyric acid, butyric acid, isovaleric acid, and valeric acid, are products of carbohydrate fermentation by gut microbiota. Figure 5A–G demonstrates the alterations in SCFA concentrations in the intestinal contents after SWEPFT administration. The quantities of acetic acid, propionic acid, isobutyric acid, butyric acid, isovaleric acid, valeric acid, and total SCFAs exhibited substantial increases in both the SWEPFT-L and SWEPFT-H cohorts compared to those in the Model cohort (*p* < 0.05). Specifically, the isobutyric acid concentration in the SWEPFT-H cohort displayed a marked reduction compared to that in the SWEPFT-L and MET cohorts (*p* < 0.05) (Figure 5C). In contrast, the butyric acid concentration was notably elevated in the SWEPFT-H cohort versus the SWEPFT-L and MET cohorts (*p* < 0.05) (Figure 5D). Moreover, the isovaleric acid and valeric acid levels were substantially enhanced in the SWEPFT-H cohort versus the SWEPFT-L cohort (*p* < 0.05) (Figure 5E,F).

The link between bacterial genera and SCFAs was examined through a correlation heatmap analysis. As depicted in Figure 5H, *Butyricimonas* demonstrated strong positive links to acetic acid, propionic acid, isobutyric acid, butyric acid, isovaleric acid, valeric acid, and total SCFAs (*p* < 0.05). *Barnesiella* and *Parabacteroides* were markedly positively linked to acetic acid (*p* < 0.05); conversely, *Anaerotruncus*, *Lactococcus*, *Allobaculum*, *Macellibacteroides*, and *Faecalibaculum* showed marked negative links to acetic acid (*p* < 0.05). *Oscillibacter* and *Peptococcus* were markedly positively linked to butyric acid, valeric acid, and isovaleric acid (*p* < 0.05). In contrast, *Anaerotruncus*, *Bradyrhizobium*, *Holdemanella*, *Terrimicrobium*, and *unidentified_Erysipelotrichaceae* exhibited significant negative correlations with butyric acid, isovaleric acid, and valeric acid (*p* < 0.05). To further elucidate the relationship between hypoglycemic parameters and SCFAs, a Mantel test was performed (Figure 5I). The analysis revealed that hypoglycemic parameters such as FBG, AUC of OGTT, serum GSP, HOMA-IS, HOMA-IR, and HOMA-β were markedly linked to acetic acid, propionic acid, isobutyric acid, butyric acid, isovaleric acid, and valeric acid (*p* < 0.001).

### 3.6. Effects of Link Between Differential Bacterial Genera and Parameters of Hypoglycemia

**Figure 6:**
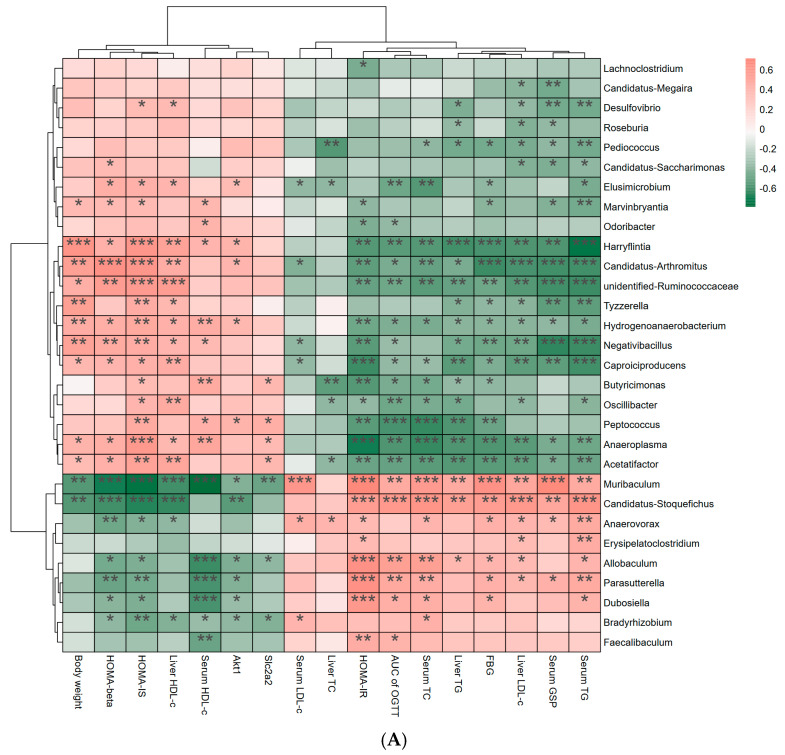

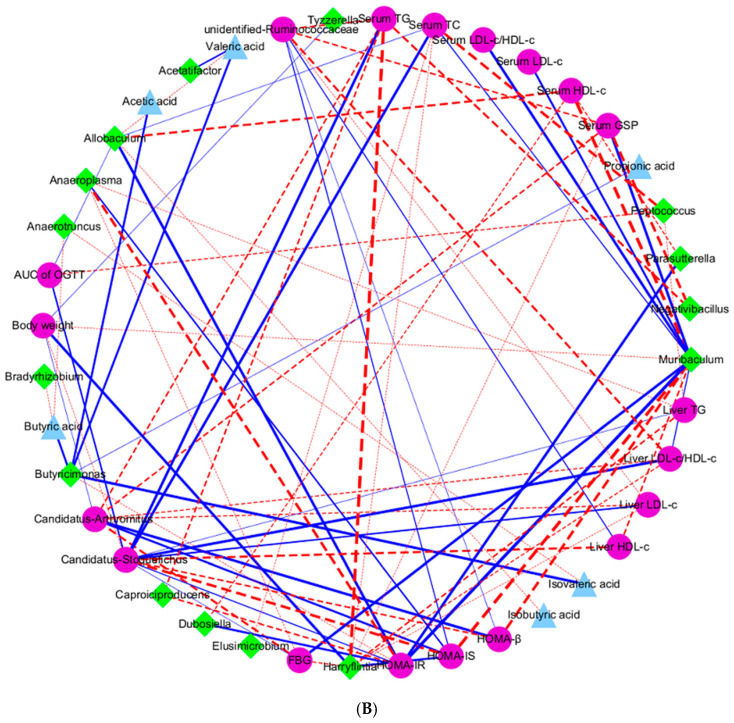
Hierarchical clustering analysis (**A**) and correlation network visualization (**B**) Spearman correlation between hypoglycemic parameters and representative bacterial genera. In (**A**), salmon indicates positive correlations, while seagreen indicates negative correlations, with color depth reflecting the strength of correlation. * *p* < 0.05, ** *p* < 0.01, and *** *p* < 0.001 indicate the significant relationship between hypoglycemic parameters and representative bacterial genera. In (**B**), fuchsia, lime, and lightskyblue indicate hypoglycemic parameters, bacterial genera, and SCFAs, respectively. Blue solid lines represent r > 0.6 with adjusted *p* < 0.01, and red dotted lines represent r < −0.6 with adjusted *p* < 0.01.

This investigation analyzed the link between characteristic differential bacterial genera and hypoglycemic indicators after SWEPFT treatment (Figure 6A). *Oscillibacter* demonstrated substantial positive associations with liver HDL-c and HOMA-IS (*p* < 0.05), whereas it displayed negative links to serum TG, liver LDL-c, liver TG, serum TC, AUC of OGTT, HOMA-IR, and liver TC (*p* < 0.05). *Muribaculum* displayed marked positive correlations with FBG, AUC of OGTT, HOMA-IR, serum GSP, serum TC, serum TG, liver LDL-c, liver TG, and serum LDL-c (*p* < 0.05), and marked negative correlations with *Akt1*, *Slc2a2*, serum HDL-c, liver HDL-c, HOMA-IS, HOMA-β, and BW (*p* < 0.05). Based on Figure 6B, parameters exhibiting correlation coefficients |r| ≥ 0.55 were identified. *Peptococcus* displayed marked negative associations with serum TC, OGTT AUC, and liver TG (r = −0.636, −0.582, and −0.569, respectively). Notably, *Muribaculum* revealed substantial negative associations with serum HDL-c, HOMA-IS, HOMA-β, liver HDL-c, and BW (r = −0.787, −0.742, −0.693, −0.594, and −0.561, respectively), while demonstrating significant positive associations with the liver LDL-c/HDL-c ratio, serum TC, serum LDL-c, FBG, serum GSP, and HOMA-IR (r = 0.573, 0.585, 0.614, 0.594, 0.655, 0.679, 0.693, and 0.717, respectively). The analysis revealed that *Candidatus_Arthromitus*, *Anaeroplasma*, *Candidatus_Stoquefichus*, and *Harryflintia* demonstrated significant associations with various hypoglycemic parameters. Specifically, *Anaeroplasma* displayed negative associations with FBG and OGTT AUC (r = −0.562 and −0.568, respectively), whereas *Candidatus_Stoquefichus* exhibited positive associations with FBG and OGTT AUC (r = 0.57 and 0.599, respectively).

## 4. Discussion

T2DM is a prevalent endocrine and metabolic disorder worldwide, posing significant health risks, including increased morbidity, especially in aging populations, microangiopathy-induced diabetic retinopathy, and other complications that cannot be overlooked [22]. Sustained intake of high-sugar and high-fat diets induces a range of adverse effects, such as elevated FBG levels, abnormal OGTT, and pancreas islet dysfunction. Furthermore, chronic hyperglycemia exacerbates fat deposition in liver tissue, leading to liver inflammation and tissue injury [23]. Therefore, unhealthy lifestyle habits, including high-sugar and high-fat diets, not only disrupt the normal regulation of blood glucose levels but also impair liver function, accelerating progression of T2DM and related disorders. Elevated glucose concentrations impede cellular uptake and metabolism of glucose and alternative energy sources. Consequently, these energy substrates are excreted in the urine rather than being used for maintaining normal physiological functions, which ultimately results in rapid weight loss in T2DM mice [24]. In this investigation, after four weeks of intervention with varying doses of SWEPFT in T2DM mice, a significant trend toward weight recovery was observed in the SWEPFT-L and SWEPFT-H cohorts. These findings indicate that SWEPFT facilitates glucose uptake and energy substrate metabolism in T2DM mice, reduces their excretion, and alleviates the abnormal weight loss associated with this condition. Notably, SWEPFT-H’s effect on BW recovery was more pronounced than that of SWEPFT-L. Specifically, throughout weeks two and four of MET administration, there were no substantial variations in BW between the MET and Model cohorts, which could potentially be attributed to MET’s effects on gastrointestinal absorption and energy substrate processing, which might have hindered a significant BW improvement.

FBG remains the most commonly used measure for assessing T2DM and is widely employed in the initial screening and diagnosis of the disease [25]. In this study, the blood glucose levels in the T2DM mice were markedly diminished after SWEPFT intervention at both 2 and 4 weeks, with FBG levels approaching those of the MET cohort. At the 4-week mark, the FBG level in the SWEPFT-H cohort was markedly diminished compared to that in the SWEPFT-L cohort. These preliminary findings suggest that both SWEPFT-L and SWEPFT-H exert hypoglycemic effects on T2DM mice, with SWEPFT-H demonstrating superior efficacy in reducing blood glucose levels. In clinical research, while FBG serves as a standard initial screening and diagnostic tool for T2DM, it only captures blood glucose status at an isolated moment and exhibits natural variations. Therefore, a diagnosis based exclusively on FBG measurements risks potential diagnostic inaccuracies, especially near critical threshold values. To overcome these constraints and enhance diagnostic accuracy, investigations have identified alternative detection approaches. For example, Jiao et al. [26] combined several parameters, including OGTT, AUC of OGTT, and serum GSP, to establish a comprehensive diagnostic system, providing enhanced reliability for early T2DM identification and intervention. The OGTT evaluates glucose regulation capabilities following glucose consumption, emphasizing the physiological response to glucose variations [27]. The OGTT diagnostic standards primarily assess blood glucose deviations from normal ranges at specific intervals, while the AUC of OGTT provides significant reference data by analyzing glucose measurements across four distinct points. Furthermore, serum GSP demonstrates greater stability against short-term glucose fluctuations, presenting a more accurate indication of glycemic status over 2–3 weeks [28]. In this investigation, the OGTT curves for the SWEPFT-L and SWEPFT-H cohorts were positioned between those of the Normal and Model cohorts. Furthermore, the AUC of OGTT and serum GSP data indicated that both SWEPFT-L and SWEPFT-H markedly improved hyperglycemic symptoms in the T2DM mice. Recent research indicates that compromised pancreatic β-cell functionality serves as the fundamental trigger for T2DM development, with functional decline correlating to disease severity [29]. Notably, chronic dysfunction of pancreas islet β-cells can trigger and intensify insulin resistance patterns. Consequently, monitoring pancreas islet β-cell performance remains crucial for T2DM prevention, control, and therapeutic interventions. HOMA represents an established approach for pancreas islet assessment, incorporating FBG measurements and insulin serum levels to analyze insulin resistance, pancreas islet β-cell capability, and insulin responsiveness through HOMA-IR, HOMA-β, and HOMA-IS parameters [30]. The experimental findings demonstrated that post-SWEPFT administration, substantial enhancements emerged in both the HOMA-β and HOMA-IR metrics, with the SWEPFT-H cohort displaying notably superior HOMA-IS outcomes. Nevertheless, the comparison between the SWEPFT-L and Model cohorts revealed no substantial variations, indicating that SWEPFT-L administration failed to produce meaningful improvements in insulin sensitivity among the T2DM mice.

Additionally, pancreatic islet dysfunction triggers elevated endogenous triglyceride production within hepatic tissue, diminished glucose uptake in peripheral regions, and increased lipolytic activity. Subsequently, fatty acids circulate toward hepatic tissue, undergoing conversion into triglycerides for storage, thereby intensifying dyslipidemia manifestations [31]. This investigation revealed pathological indicators of disrupted lipid metabolism and hepatic damage in T2DM mice. Notable findings included substantial increases in TC, TG, and LDL-c concentrations in both serum and liver samples, accompanied by markedly decreased HDL-c levels, suggesting severe metabolic lipid disruption. Research by Zou et al. [32] demonstrated that the ratio between LDL-c and HDL-c functions as an autonomous indicator for nonalcoholic fatty liver disease, T2DM, and related metabolic conditions, exhibiting superior predictive capability compared to individual HDL-c or LDL-c measurements. Histological examination revealed disordered hepatic cord arrangement, likely due to hepatocyte injury, edema, or inflammation, with numerous lipid vacuoles occupying hepatocyte space and impairing liver function. This further underscores the strong association between lipid metabolism disturbances in T2DM and liver tissue damage. Following the SWEPFT-L and SWEPFT-H treatments, these indicators and pathological features were notably improved, indicating that SWEPFT can markedly ameliorate lipid metabolism disorders in T2DM mice. Research indicates that *Akt1* is capable of regulating *Slc2a2* transcription in liver tissues, strengthening the liver’s capacity to uptake glucose from the circulation, facilitating cellular energy metabolism, and optimizing glucose utilization efficiency, consequently diminishing hyperglycemia [33]. Therefore, investigating *Akt1* and *Slc2a2* transcriptional activity within liver tissue holds substantial importance. Our analysis revealed that *Akt1* and *Slc2a2* demonstrated notably elevated transcription levels in the SWEPFT-H treatment cohort when compared to SWEPFT-L administration. The findings indicate that SWEPFT-H exhibits superior efficacy in stimulating *Akt1* and *Slc2a2* transcription throughout liver tissue, thus facilitating the amelioration of hyperglycemic conditions in T2DM mice.

Gut microorganism equilibrium exhibits strong connections to host physiological mechanisms, specifically regarding glucose homeostasis regulation [34]. Distinct variations are observed when comparing intestinal bacterial communities between healthy subjects and those diagnosed with T2DM. For instance, in patients with T2DM, the Firmicutes/Bacteroidetes ratio is notably elevated [35]. In this study, at the phylum level, the Firmicutes/Bacteroidetes ratio was markedly reduced after SWEPFT treatment, approaching the levels seen in the Normal cohort. This suggests that both SWEPFT-L and SWEPFT-H successfully normalized the gut microbial structure at the phylum level in T2DM mice, aligning with observations documented by Yang et al. [36]. Regarding the genus-level analysis, microbial classification and functionality exhibit greater complexity and variation. Research by Chai et al. demonstrated significant enhancement in *Intestinimonas* populations following *Polygonatum sibiricum* saponin administration in T2DM mice, corresponding to the experimental outcomes reported herein [37]. Additionally, SWEPFT-H markedly elevated *Ruminiclostridium* abundance in the T2DM mice. This finding mirrors previous research where 6,8-guanidyl luteolinquinone-chromium coordination modulated the intestinal microbiota in T2DM mice, elevating the *Ruminiclostridium* abundance and improving hyperglycemia symptoms [25]. This study has confirmed the overall beneficial regulatory effects of SWEPFT on the intestinal microbiota. Additionally, the flavonoids and isoflavonoids in SWEPFT share the important ability to regulate the intestinal microbiota. That is, both can serve as fermentation substrates for the intestinal microbiota, thereby increasing the abundance of beneficial bacteria and optimizing the microbial community [38]. They also have specific effects based on their chemical structures. For instance, Liao et al. found that nobiletin can improve secondary bile acid metabolism by regulating the intestinal microbial community, thereby achieving hyperglycemia [39]. Formononetin reduces the body’s inflammatory response by increasing the relative abundance of *Clostridium aldenense* and *Eubacterium plexicaum*, thereby maintaining the integrity of the intestinal mucosa [40]. Flavonoids and isoflavonoids jointly contribute to the overall effects. It is worth noting that SWEPFT’s influence on the microbial community can be divided into direct and indirect effects. Regarding the former, flavonoids, isoflavonoids, and possibly soluble dietary fiber in SWEPFT form a unique substrate combination. In the colon, they can be metabolized as exclusive substrates by specific bacterial genera, thereby directly driving the proliferation of these bacteria [17]. In addition, these substrates may also act like “prebiotics”, serving as regulatory signals to influence the abundance and community of the microbial. Therefore, SWEPFT is very likely to be able to reshape the intestinal microecology through this direct input of active ingredients. In terms of indirect effects, some components in SWEPFT may first exert pharmacological effects in the small intestine. For instance, the flavonoid nobiletin has been reported to display certain inhibitory activities against α-glucosidase or α-amylase [41]. If such inhibition exists, it will lead to delayed digestion of starch and disaccharides in the daily diet, increasing the amount entering the colon. This is similar to the effect of acarbose, which indirectly affects the microbiota by altering the availability of dietary components in the host. Xiao et al. combined *Scutellaria baicalensis* and *Coptis chinensis* in T2DM rats and found that SCFA-producing microbiota, including *Ruminiclostridium*, were markedly enriched [42]. SCFAs, generated by gut microorganisms via fermentation of indigestible carbohydrates with chains containing 1-6 carbon atoms, encompass acetic, propionic, isobutyric, butyric, isovaleric, and valeric acid. These SCFAs are absorbed through the intestinal epithelium and function as energy substrates. SCFA elevation has demonstrated the capability to enhance beneficial bacterial growth, decreasing detrimental bacterial populations, and sustaining microbial equilibrium in the intestine. Through the activation of internal and external signaling pathways within intestinal mucosal cells, three primary SCFAs—acetic, propionic, and butyric acids—lead to advantageous outcomes for epithelial and immune cells in the intestinal system [43]. As the most abundant SCFAs in the intestine, acetic acid serves a pivotal component in lipid and cholesterol synthesis, while in the liver, it reduces lipid accumulation and improves metabolic function [44]. Propionic acid, primarily absorbed by the liver, helps maintain pancreas islet β-cell function by inhibiting apoptosis and enhancing their sensitivity [45]. Butyric acid enhances glucose uptake in muscle and adipose tissue, thus regulating blood glucose levels. In this investigation, both SWEPFT and MET increased the SCFA levels compared to those in the Model group, but they influenced the gut microbial community differently. The main pharmacological effect of MET lies in its activation of the AMPK pathway to improve insulin sensitivity [46]. The mechanism by which it increases SCFAs is considered indirect and may cause more host-derived carbohydrates to become substrates for fermentation by microbiota by altering intestinal transport time, bile acid metabolism, or the intravascular osmotic pressure [47,48]. These substrates tend to promote acetic acid production [49], which is consistent with the fact that the acetic acid level in the MET group was significantly higher than that in the SWEPFT-L and SWEPFT-H groups in this study. In contrast, flavonoids, isoflavonoids, and, possibly, soluble dietary fiber in SWEPFT form a unique substrate combination. In this study, SWEPFT-H significantly increased the relative abundance of *Oscillibacter*. It has been reported that *Oscillibacter* increases the level of butyric acid in the intestine by metabolizing these substrates [50]. This suggests that SWEPFT may have encouraged the bacterial communities capable of fermenting these substrates and producing butyric acid by providing such complex plant-based substrates. Therefore, although both increase the level of SCFAs, their potential regulatory mechanisms are different. MET may indirectly affect substrate availability by regulating host physiological functions and promoting the production of SCFAs mainly composed of acetic acid; SWEPFT, on the other hand, may promote a bacterial community that is more inclined to produce butyric acid through using the phytochemicals it provides as direct substrates. Numerous studies have indicated that certain bacterial genera can modulate SCFA production; for example, Piovani et al. identified *Butyricimonas* as a beneficial genus producing SCFAs [51]. Deng et al. reported that SCFA production in T2DM mice was closely linked to the relative abundance of *Odoribacter* [52]. Additionally, Gu et al. showed that increased SCFA levels enhance the secretion of downstream hormones, improving hyperglycemia-related markers and alleviating T2DM symptoms [53]. These observations align with this investigation’s findings, in which the *Butyricimonas* and *Odoribacter* abundances were markedly positively linked to SCFA levels, and SCFAs were associated with several hypoglycemic parameters like FBG, serum GSP, and HOMA-IR.

To examine the effects of SWEPFT on hyperglycemia through modulation of the intestinal microbiota, a hierarchical cluster analysis was executed to assess the link between bacterial genera and hypoglycemic indicators. Zhang et al. examined the impact of calorie restriction on the intestinal microbiota in T2DM rats, revealing that calorie restriction not only regulated the microbial community but also specifically elevated the abundance of anti-inflammatory bacteria like *Oscillibacter*, thus reducing inflammation markers like IL-6 and TNF-α [54]. Fassatoui et al. analyzed the gut microbiota profiles of diabetic and healthy Tunisian individuals, finding that the *Oscillibacter* abundance was markedly diminished in the T2DM populations [55]. Shen et al. demonstrated that gingerol-enriched ginger could reduce the *Muribaculum* abundance in T2DM rats [56]. In this study, *Muribaculum* was found to exhibit a marked positive link to FBG, serum GSP, serum TC, HOMA-IR, and serum LDL-c, confirming its association with T2DM. Additionally, Mo et al. identified *Butyricimonas* as a key bacterium utilizing dietary fiber, with significant correlations to LDL-c, HDL-c, and other metabolic indicators [57]. In this research, *Butyricimonas* displayed a significant positive link to serum HDL-c, consistent with Mo et al.’s findings [57]. Furthermore, *Anaeroplasma* and *Candidatus_Stoquefichus* were strongly linked to the FBG and AUC of OGTT, aligning with the observations of Chen et al. [58] and Huang et al. [59]. The study also found that *Candidatus_Arthromitus*, *Anaeroplasma*, *Candidatus_Stoquefichus*, and *Harryflintia* exhibited robust correlations with various hypoglycemic indicators, indicating their possible utility as intestinal biomarkers for additional exploration of T2DM development and advancement.

These findings collectively demonstrate that both SWEPFT-L and SWEPFT-H treatments successfully ameliorate hyperglycemic manifestations in T2DM mice. The enhancements in BW, FBG, AUC of OGTT, HOMA-IS, serum TC, liver TG, liver LDL-c, liver HDL-c, and relative transcription levels of *Akt1* and *Slc2a2*, along with butyric acid, valeric acid, and isovaleric acid concentrations, were markedly more substantial in the SWEPFT-H cohort versus the SWEPFT-L cohort. This research further substantiates the critical role of SWEPFT in alleviating T2DM symptoms through modulation of the intestinal microbial community.

## 5. Conclusions

In this investigation, a T2DM mouse model was generated by utilizing an HSHF diet plus STZ intraperitoneal administration. It was revealed that varying concentrations of SWEPFT led to marked improvements in hyperglycemia-associated parameters in these T2DM mice. Compared to the low-dose treatment, high doses of SWEPFT demonstrated superior efficacy regarding multiple indicators: BW, FBG, OGTT curve area, HOMA-IS, TC in serum, hepatic TG, liver-specific LDL-c, HDL-c levels, *Akt1* and *Slc2a2* transcription patterns, as well as butyric, valeric, and isovaleric acid concentrations. Additionally, SWEPFT administration induced substantial alterations in the T2DM mouse gut microbial composition, characterized by a marked decrease in the Firmicutes to Bacteroides proportion at the phylum level and elevated populations of beneficial bacteria including *Intestinimonas* and *Ruminiclostridium* genera, which was coupled with increased cecal SCFA production. Additionally, this study identified a strong correlation between *Candidatus_Arthromitus*, *Anaeroplasma*, *Candidatus_Stoquefichus*, and *Harryflintia* with multiple hypoglycemic indicators, suggesting their role as bacterial markers in T2DM development. These observations demonstrate SWEPFT’s promising therapeutic capabilities in T2DM treatment, establishing groundwork for subsequent research and medical implementations.

## Figures and Tables

**Table 1:** List of all primers used for qPCR.

PRIMER NAME	Forward Primer (5′–3′)	Reverse Primer (5′–3′)
*Actb*	TGTCCACCTTCCAGCAGATGT	AGCTCATAACAGTCCGCCTAGA
*Akt1*	ACTCATTCCAGACCCACGAC	CCGGTACACCACGTTCTTCT
*Slc2a2*	TACGGCAATGGCTTTATC	CCTCCTGCAACTTCTCAAT

## Data Availability

The original contributions presented in this study are included in the article/Supplementary Materials. Further inquiries can be directed to the corresponding authors.

## Supplementary Materials

The following supporting information can be downloaded at https://www.mdpi.com/article/10.3390/foods15010143/s1. Table S1. The primary chemical constituents of SWEPFT.
