# Supplementary material for: Supernatants from Water Extraction—Ethanol Precipitation of Fagopyrum tararicum Seeds Enhance T2DM Management in Mice by Regulating Intestinal Microbial Communities
Source: Foods. 2026 Jan 2;15(1):143. doi: 10.3390/foods15010143 (PMC12785855; doi:10.3390/foods15010143)
Supplement: Supplementary file 1 [file foods-15-00143-s001.zip › foods-4023767-supplementary.pdf]

Supplementary Tables

Supplementary Table S1 The primary chemical constituents of SWEPFT

index	MS2_name	RT	m/z	JY-2	Class	CAS
M401T514_NEG	Nobiletin	8.562	401.1304886	3244706684	Flavonoids	478-01-3
M237T237_NEG	5-hydroxyflavone	3.95	237.061709	1713425498	Flavonoids	71802-04-5
M391T394_NEG	Glabrol	6.56	391.2092467	580774809.8	Flavonoids	59870-65-4
M315T493_NEG	Quercetin 3'-methyl ether	8.209	315.0490665	374279805.1	Flavonoids	480-19-3
M223T258_POS	Flavone	4.305	223.0826825	308142529	Flavonoids	525-82-6
M593T570_NEG	Poncirin	9.504	593.1942425	244037080.7	Flavonoids	14941-08-3
M289T438_NEG	Catechin	7.299	289.0681357	239738818.8	Flavonoids	154-23-4
M301T122_2_NEG	Morin	2.028	301.0568406	229547096.6	Flavonoids	480-16-0
M253T274_NEG	Chrysin	4.559	253.0720242	227387271.9	Flavonoids	480-40-0
M453T50_POS	Hecogenin	0.836	453.3052903	89236590.01	Flavonoids	115921-09-0
M221T358_NEG	Flavone	5.968	221.0666775	52606712.62	Flavonoids	525-82-6
M239T441_POS	5-hydroxyflavone	7.356	239.1023466	42487259.42	Flavonoids	71802-04-5
M465T461_POS	Hyperoside	7.675	465.118382	27335477.69	Flavonoids	482-36-0
M457T489_NEG	Epigallocatechin gallate	8.151	457.0969577	27272024.38	Flavonoids	989-51-5
M579T570_NEG	Naringin	9.504	579.1801416	25088947.97	Flavonoids	10236-47-2
M287T300_POS	4',5-dihydroxy-7-methoxyflavanone	4.998	287.1025738	22326286.72	Flavonoids	2957-21-3
M289T464_POS	Eriodictyol	7.73	289.0712283	20483220.35	Flavonoids	552-58-9
M271T499_NEG	Quercetagetin	8.315	271.0230066	19861674.87	Flavonoids	1251-84-9
M437T442_POS	Phlorizin	7.368	437.1664499	15683335.45	Flavonoids	60-81-1
M287T25_NEG	Eriodictyol	0.411	286.9765653	12945522.05	Flavonoids	552-58-9
M269T46_3_NEG	Apigenin	0.771	269.2490252	4902717.188	Flavonoids	520-36-5
M271T695_POS	Apigenin	11.576	271.0848636	3054866.356	Flavonoids	520-36-5
M301T64_1_NEG	Quercetin	1.064	300.9798814	2571905.662	Flavonoids	117-39-5
M317T504_POS	Quercetin 3'-methyl ether	8.408	317.0528039	1316316.486	Flavonoids	480-19-3
M577T42_POS	Procyanidin a2	0.695	577.126262	710036.6616	Flavonoids	41743-41-3
M89T378_5_NEG	L-(+)-lactic acid	6.305	89.02344627	1.29704E+11	Indoles and derivatives	87-51-4
M144T437_1_NEG	Indole-3-carboxaldehyde	7.289	144.0295674	6110860158	Indoles and derivatives	487-89-8
M158T356_2_NEG	Indole-3-butyric acid	5.929	158.0817209	5863197239	Indoles and derivatives	133-32-4
M203T395_2_NEG	Tryptophan	6.59	203.0823877	5729867643	Indoles and derivatives	153-94-6
M188T395_2_POS	Indolelactic acid	6.591	188.0721239	3903856490	Indoles and derivatives	7417-65-4
M160T411_NEG	3-(2-hydroxyethyl)indole	6.847	160.0610869	1879951731	Indoles and derivatives	526-55-6
M204T318_NEG	5-methoxyindoleacetate	5.303	204.0664284	987963331.3	Indoles and derivatives	3471-31-6
M169T63_POS	Norharmane	1.058	169.0754998	899465783	Indoles and derivatives	244-63-3
M144T395_2_POS	3-(2-hydroxyethyl)indole	6.591	144.0820161	782393753.2	Indoles and derivatives	526-55-6
M187T407_POS	L-tryptophanamide	6.789	187.1092913	580063265.5	Indoles and derivatives	20696-57-5
M160T281_2_POS	Bufotenine	4.686	160.0995693	566947635.1	Indoles and derivatives	487-93-4
M146T395_POS	Indole-3-carboxaldehyde	6.591	146.0612537	472147273.1	Indoles and derivatives	487-89-8
M144T322_POS	N-methyltryptamine	5.374	144.0808943	437364695.4	Indoles and derivatives	61-49-4
M160T377_NEG	Indole-3-carboxylic acid	6.28	160.0610235	427317089.6	Indoles and derivatives	771-50-6
M160T70_POS	Serotonin	1.159	160.0757278	424120689.4	Indoles and derivatives	50-67-9
M186T348_POS	Indole-3-butyric acid	5.805	186.1142424	401656904.1	Indoles and derivatives	133-32-4
M227T493_POS	D-tryptophan	8.215	227.0812456	335514081.1	Indoles and derivatives	153-94-6
M242T267_NEG	Etodolac	4.443	242.1762474	295148913.6	Indoles and derivatives	41340-25-4
M217T533_NEG	N-Acetylserotonin	8.878	217.0831257	294290827.4	Indoles and derivatives	1210-83-9
M174T531_1_NEG	Indoleacetic acid	8.852	174.0407652	293317951.1	Indoles and derivatives	87-51-4
M190T407_1_NEG	5-hydroxyindoleacetate	6.788	190.071863	263058832.5	Indoles and derivatives	54-16-0
M186T527_NEG	Indolelactic acid	8.778	186.0405251	229309536.6	Indoles and derivatives	7417-65-4
M202T521_NEG	Indole-3-pyruvic acid	8.676	202.0718929	227688421.9	Indoles and derivatives	392-12-1
M175T215_POS	Indole-3-acetamide	3.587	175.0867073	216000610.4	Indoles and derivatives	879-37-8
M231T389_2_NEG	Melatonin	6.479	231.1350259	201377266.7	Indoles and derivatives	73-31-4
M323T37_NEG	Dolasetron	0.622	323.1543025	193200837.7	Indoles and derivatives	115956-12-2
M175T214_NEG	Serotonin	3.571	175.1123675	185218870	Indoles and derivatives	50-67-9
M294T589_POS	Ondansetron	9.812	294.1592028	170083023.3	Indoles and derivatives	99614-02-5
M203T431_NEG	D-tryptophan	7.177	203.0671677	149411376	Indoles and derivatives	153-94-6
M231T166_POS	Pindolol	2.767	231.1492255	138997609.4	Indoles and derivatives	13523-86-9
M132T395_2_POS	3-methylindole	6.591	132.0819601	138141841.2	Indoles and derivatives	83-34-1
M155T361_NEG	Indole-3-acetonitrile	6.012	155.0013841	106386531.8	Indoles and derivatives	771-51-7
M157T283_POS	Indole-3-acetonitrile	4.711	157.0761258	85148622.22	Indoles and derivatives	771-51-7
M204T490_POS	Indole-3-pyruvic acid	8.171	204.0884721	82448717.74	Indoles and derivatives	392-12-1
M203T328_NEG	Psilocin	5.459	203.1286911	79773641.11	Indoles and derivatives	520-53-6
M219T124_POS	N-Acetylserotonin	2.065	219.1128784	68530841.51	Indoles and derivatives	1210-83-9
M176T438_POS	Indoleacetic acid	7.302	176.0932776	65991683.7	Indoles and derivatives	87-51-4
M247T342_2_NEG	6-hydroxymelatonin	5.705	247.1125182	64311491.54	Indoles and derivatives	2208-41-5
M167T414_NEG	Norharmane	6.893	167.0821251	56543992.24	Indoles and derivatives	244-63-3
M257T245_POS	19-hydroxytestosterone	4.086	257.1857942	54429353.94	Indoles and derivatives	2126-37-6
M320T68_POS	Dimebon	1.138	320.2329255	50845005.77	Indoles and derivatives	3613-73-8
M142T394_NEG	Indoleacrylic acid	6.569	142.0866511	47115067.78	Indoles and derivatives	29953-71-7
M158T282_NEG	Indole-3-acetaldehyde	4.696	158.0817626	41498878.58	Indoles and derivatives	2591-98-2
M173T395_2_NEG	Indole-3-acetamide	6.579	173.0814942	38171591.81	Indoles and derivatives	879-37-8
M161T237_POS	Tryptamine	3.942	161.1073451	36718707.95	Indoles and derivatives	61-54-1

M233T33_NEG	5-methoxytryptophan	0.553	233.085701	31940916.23	Indoles and derivatives	2504-22-5
M219T155_POS	N-acetyl-5-hydroxytryptamine	2.575	219.1125572	27990195.05	Indoles and derivatives	1210-83-9
M189T514_NEG	5-methoxytryptamine	8.562	189.0879269	24511315.4	Indoles and derivatives	608-07-1
M174T322_POS	Melatonin	5.362	174.1143815	16811482.26	Indoles and derivatives	73-31-4
M201T378_POS	Camalexin	6.299	201.0721266	13861091.2	Indoles and derivatives	135531-86-1
M191T513_2_POS	5-methoxytryptamine	8.55	191.105552	12394432.25	Indoles and derivatives	608-07-1
M402T432_POS	Fumitremorgin c	7.196	402.163402	10179190.3	Indoles and derivatives	118974-02-0
M146T141_NEG	Isatin	2.354	146.0103332	8262750.407	Indoles and derivatives	91-56-5
M288T907_POS	Rutaecarpine	15.112	288.0912221	6359117.742	Indoles and derivatives	84-26-4
M182T944_POS	Zolmitriptan	15.729	182.0838574	6141293.736	Indoles and derivatives	139264-17-8
M242T98_POS	Mefenamic acid	1.635	242.1385612	1766931841	Isoflavonoids	4044-00-2
M267T62_NEG	Formononetin	1.035	267.2335336	238280845	Isoflavonoids	485-72-3
M201T415_NEG	Glabridin	6.914	201.08786	234541835.1	Isoflavonoids	59870-68-7
M253T28_NEG	Daidzein	0.46	253.03604	224981364.6	Isoflavonoids	486-66-8
M475T312_NEG	Ononin	5.203	475.1381885	179412964.7	Isoflavonoids	486-62-4
M305T507_1_NEG	Genistein	8.447	305.0233308	171721334.6	Isoflavonoids	446-72-0
M240T321_NEG	Mefenamic acid	5.357	240.0865341	75266498.59	Isoflavonoids	4044-00-2
M423T316_NEG	Licoricidin	5.273	423.2871717	70219447.86	Isoflavonoids	30508-27-1
M335T202_NEG	Psoralidin	3.369	335.0707056	63151169.28	Isoflavonoids	18642-23-4
M283T27_1_NEG	Biochanin a	0.45	283.0469451	26137492.39	Isoflavonoids	491-80-5
M284T481_NEG	Tectorigenin	8.019	284.0546312	25422369.86	Isoflavonoids	548-77-6
M393T487_NEG	Rotenone	8.11	393.1521342	14570882.15	Isoflavonoids	83-79-4
M241T323_POS	Rotenone	5.387	241.0970426	12877568.72	Isoflavonoids	83-79-4
M437T43_NEG	5-o-methyllicoricidin	0.711	437.241347	15689.20678	Isoflavonoids	129314-37-0
M209T490_3_NEG	Sinapyl alcohol	8.162	209.0663686	14217138981	Phenols	537-33-7
M168T158_POS	Phenylephrine	2.637	168.1019905	12543074383	Phenols	59-42-7
M173T587_NEG	4-bromophenol	9.782	172.9575548	3784727000	Phenols	106-41-2
M193T254_NEG	4-hexylresorcinol	4.241	193.1231422	2964824066	Phenols	136-77-6
M109T76_NEG	Pyrocatechol	1.275	109.0035844	2599611851	Phenols	120-80-9
M184T262_POS	Epinephrine	4.358	184.1081783	1111157218	Phenols	51-43-4
M208T322_POS	Terbutaline	5.362	208.1501425	982164900.6	Phenols	23031-25-6
M151T363_2_NEG	3-hydroxyphenylacetic acid	6.045	151.0606295	915456270.8	Phenols	621-37-4
M154T91_POS	Dl-octopamine	1.515	154.0863659	849050127.1	Phenols	104-14-3
M149T150_NEG	Homogentisic acid	2.494	149.0239214	783312544.3	Phenols	102-32-9
M198T88_POS	Metanephrine	1.47	198.1125023	601689790.3	Phenols	5001-33-2
M111T255_POS	Hydroquinone	4.255	111.0556355	454638809.9	Phenols	123-31-9
M240T559_POS	Isoetharine	9.309	240.1745654	397882149.6	Phenols	530-08-5
M139T56_POS	4-hydroxyphenylethanol	0.938	139.0866888	337915524.6	Phenols	501-94-0
M152T228_NEG	Dopamine	3.792	152.0711639	334008825.5	Phenols	62-31-7
M137T363_POS	Dobutamine	6.058	137.0470762	321261594	Phenols	34368-04-2
M211T277_POS	Sinapyl alcohol	4.619	211.1077771	298923021.6	Phenols	537-33-7
M150T54_NEG	Acetaminophen	0.906	150.0318202	297376257.8	Phenols	103-90-2
M183T214_NEG	3,4-dihydroxymandelic acid	3.569	183.0062699	293132240.4	Phenols	775-01-9
M125T213_NEG	1,2,3-benzenetriol	3.55	125.0348718	270563652.5	Phenols	87-66-1
M336T417_NEG	Dobutamine	6.946	336.1304602	259222585.9	Phenols	34368-04-2
M166T154_NEG	Phenylephrine	2.559	166.0869238	242627986.2	Phenols	59-42-7
M109T117_NEG	Hydroquinone	1.943	109.0286411	210177198	Phenols	123-31-9
M93T323_NEG	Phenol	5.378	93.03373915	202418035.7	Phenols	87-66-1
M220T193_NEG	Isoetharine	3.21	220.1468509	146038017.2	Phenols	530-08-5
M109T44_NEG	Resorcinol	0.736	109.0287558	132752265.8	Phenols	108-46-3
M168T114_NEG	Norepinephrine	1.899	168.06609	129610363.7	Phenols	51-41-2
M293T193_NEG	[6]-gingerol	3.219	293.1763455	114937335.9	Phenols	58253-27-3
M308T250_POS	Dihydrocapsaicin	4.159	308.2218192	99258120.62	Phenols	19408-84-5
M168T330_POS	3-methoxytyramine	5.505	168.1132461	94642936.68	Phenols	554-52-9
M311T34_NEG	Folate	0.574	311.0783068	92705502.75	Phenols	59-30-3
M181T277_NEG	Homovanillic acid	4.613	181.1593724	86797209.05	Phenols	306-08-1
M268T445_POS	Metoprolol	7.422	268.1678072	84788626.75	Phenols	37350-58-6
M77T396_POS	Phenol	6.601	77.03971786	62420050.03	Phenols	87-66-1
M193T375_NEG	Zingerone	6.257	193.0978753	59830872.12	Phenols	122-48-5
M197T512_2_NEG	Dl-4-hydroxy-3-methoxymandelic acid	8.539	197.0063727	58081278.5	Phenols	13244-77-4
M212T74_POS	Metaproterenol	1.227	212.1472715	55816117.74	Phenols	586-06-1
M197T443_NEG	DL-Vanillylmandelic acid	7.386	197.0218308	54464333.32	Phenols	13244-77-4
M110T140_1_POS	Acetaminophen	2.34	110.0716264	46730246.51	Phenols	103-90-2
M192T308_NEG	Isoproterenol	5.129	192.1026492	45511167.93	Phenols	7683-59-2
M167T47_1_NEG	3,4-dihydroxyphenylacetic acid	0.782	166.9990647	39624813.26	Phenols	102-32-9
M277T399_POS	[6]-gingerol	6.656	277.1932451	38135355.83	Phenols	58253-27-3
M529T402_NEG	Kukoamine a	6.706	529.2999776	27506513.96	Phenols	75288-96-9
M308T53_POS	Betaxolol	0.876	308.2066777	24178211.98	Phenols	63659-18-7
M167T1016_NEG	4-hydroxymandelate	16.941	167.0094225	19198586.53	Phenols	1198-84-1
M109T512_POS	1,2,3-benzenetriol	8.537	109.0302976	18397613.57	Phenols	87-66-1
M147T322_POS	Coniferyl aldehyde	5.362	147.0440802	17531549.25	Phenols	20649-42-7
M332T523_1_NEG	Adrenaline bitartrate	8.714	332.0969406	17141098.06	Phenols	51-43-4
M336T33_POS	Bilobol	0.549	336.3107196	14165470.47	Phenols	22910-86-7

M136T409_POS	Dopamine	6.816	136.0769294	11874573.43	Phenols	62-31-7
M194T494_1_POS	Isoproterenol	8.236	194.1193554	5524127.778	Phenols	7683-59-2
M127T536_POS	1,3,5-Benzenetriol	8.932	127.0411818	5006959.714	Phenols	108-73-6
M151T633_POS	Homogentisic acid	10.548	151.0411526	3622694.815	Phenols	102-32-9
